# TMEPAI/PMEPA1 Is a Positive Regulator of Skeletal Muscle Mass

**DOI:** 10.3389/fphys.2020.560225

**Published:** 2020-11-04

**Authors:** Adam Hagg, Swati Kharoud, Georgia Goodchild, Craig A. Goodman, Justin L. Chen, Rachel E. Thomson, Hongwei Qian, Paul Gregorevic, Craig A. Harrison, Kelly L. Walton

**Affiliations:** ^1^Department of Physiology, Monash Biomedicine Discovery Institute, Monash University, Clayton, VIC, Australia; ^2^Centre for Muscle Research, Department of Physiology, The University of Melbourne, Melbourne, VIC, Australia; ^3^Baker Heart and Diabetes Institute, Melbourne, VIC, Australia; ^4^Faculty of Science, Engineering and Technology, Swinburne University of Technology, Melbourne, VIC, Australia; ^5^Australian Institute for Musculoskeletal Science, Sunshine Hospital, The University of Melbourne, St Albans, VIC, Australia; ^6^Department of Biochemistry and Molecular Biology, Monash University, Clayton, VIC, Australia; ^7^Department of Neurology, The University of Washington School of Medicine, Seattle, WA, United States; ^8^Hudson Institute of Medical Research, Clayton, VIC, Australia

**Keywords:** Gdf11, myostatin, activin, cachexia, muscle, PMEPA1, TMEPAI

## Abstract

Inhibition of myostatin- and activin-mediated SMAD2/3 signaling using ligand traps, such as soluble receptors, ligand-targeting propeptides and antibodies, or follistatin can increase skeletal muscle mass in healthy mice and ameliorate wasting in models of cancer cachexia and muscular dystrophy. However, clinical translation of these extracellular approaches targeting myostatin and activin has been hindered by the challenges of achieving efficacy without potential effects in other tissues. Toward the goal of developing tissue-specific myostatin/activin interventions, we explored the ability of transmembrane prostate androgen-induced (TMEPAI), an inhibitor of transforming growth factor-β (TGF-β1)-mediated SMAD2/3 signaling, to promote growth, and counter atrophy, in skeletal muscle. In this study, we show that TMEPAI can block activin A, activin B, myostatin and GDF-11 activity *in vitro*. To determine the physiological significance of TMEPAI, we employed Adeno-associated viral vector (AAV) delivery of a TMEPAI expression cassette to the muscles of healthy mice, which increased mass by as much as 30%, due to hypertrophy of muscle fibers. To demonstrate that TMEPAI mediates its effects via inhibition of the SMAD2/3 pathway, tibialis anterior (TA) muscles of mice were co-injected with AAV vectors expressing activin A and TMEPAI. In this setting, TMEPAI blocked skeletal muscle wasting driven by activin-induced phosphorylation of SMAD3. In a model of cancer cachexia associated with elevated circulating activin A, delivery of AAV:TMEPAI into TA muscles of mice bearing C26 colon tumors ameliorated the muscle atrophy normally associated with cancer progression. Collectively, the findings indicate that muscle-directed TMEPAI gene delivery can inactivate the activin/myostatin-SMAD3 pathway to positively regulate muscle mass in healthy settings and models of disease.

## Introduction

Myostatin, activin A, activin B, and GDF11 comprise a subgroup within the transforming growth factor-β (TGF-β) family of proteins that is of great interest in the field of skeletal muscle biology (Chen et al., 2016a). These secreted ligands negatively regulate the morphogenesis and growth of skeletal muscle by binding to activin type II receptors (ActRIIA or ActRIIB), and then recruiting and activating type I receptors (ALK4 or ALK5; Chen et al., 2016a). Activated type I receptors initiate signaling via SMAD2/3 transcription factors, which limit muscle growth via effects on both protein synthesis and protein degradation pathways. Expression of myostatin or activin A is sufficient to inhibit anabolic signaling associated with Akt/mTOR activity (Amirouche et al., 2009; Chen et al., 2014). Concurrently, SMAD2/3 activation regulates the expression of the E3 ubiquitin ligases *MuRF-1* and *Atrogin-1* (Sartori et al., 2009; Lokireddy et al., 2011; Goodman et al., 2013), which mediate ubiquitination and proteasomal degradation of myofibrillar proteins, such as myosin (Clarke et al., 2007; Fielitz et al., 2007). Ultimately, these ActRIIA/B ligands regulate genes associated with muscle protein turnover, metabolism, and sarcomere function (Chen et al., 2015, 2017) that are transcriptionally indicative of muscle wasting associated with cancer cachexia (Baker et al., 2010; Johnston et al., 2015; Rovira Gonzalez et al., 2019).

While physiological activity of these TGF-β ligands contributes to the homeostatic maintenance of muscle mass (Chen et al., 2017), elevated circulating levels are highly catabolic, inducing significant muscle wasting in murine models. For example, elevating systemic levels of myostatin, activin A or GDF11, by injecting Chinese hamster ovary (CHO) cells expressing these proteins into the quadriceps of athymic nude mice, results in a global decline in skeletal muscle mass of >20% within 12 days (Zimmers et al., 2002, 2017; Walton et al., 2019). Similar results can be observed when adeno-associated viral vectors (AAV vectors) delivered to the muscles of mice are used to elevate circulating activin A levels (Chen et al., 2014). These findings are significant because these SMAD2/3-activating ligands, particularly activin A and activin B, are elevated in many chronic conditions where muscle wasting is observed, including cancer, sepsis, lung disease, and heart disease (Michel et al., 2003; Yndestad et al., 2004, 2009; Loumaye et al., 2015, 2017). Tellingly, high activin A/B levels are predictive of adverse outcome in patients with acute respiratory failure (de Kretser et al., 2013). Thus, concerted efforts have been directed toward developing therapies that can block SMAD2/3 activity and preserve muscle mass in chronic disease.

To date, soluble activin type II receptors (sActRIIA, sActRIIB), ligand-targeting antibodies, and follistatin have proven the most efficacious therapeutic reagents in mice (Zhou et al., 2010; Winbanks et al., 2012; Latres et al., 2017), and humans (Attie et al., 2013; Campbell et al., 2017). These molecules potently antagonize signaling by activin-related ligands and reverse muscle wasting in cancer cachexia models (Li et al., 2007; Benny Klimek et al., 2010; Zhou et al., 2010). However, a recent phase II clinical trial using sActRIIB in patients with muscular dystrophy was halted when some participants experienced bleeding from mucosal surfaces (Campbell et al., 2017). This side-effect likely arises from sActRIIB inhibiting the anti-angiogenic properties of multiple TGF-β ligands (David et al., 2007) and highlights a problem associated with the use of systemically-administered pleiotropic antagonists to treat muscle wasting conditions.

An alternative approach is to identify cytoplasmic/membrane-associated skeletal muscle proteins that may inhibit SMAD2/3 activation. To this end, we recently identified *Pmepa1* as a transcriptional target of activin A signaling in skeletal muscle (Chen et al., 2014). *Pmepa1* encodes the transmembrane prostate androgen-induced (TMEPAI) protein, which is expressed in many tissues. Elevated expression of TMEPAI has been identified in tumor biopsies and cell lines derived from patients with prostate, colorectal, breast, ovarian, lung and kidney cancers (Xu et al., 2000; Rae et al., 2001; Brunschwig et al., 2003; Giannini et al., 2003). Evidence indicates that TMEPAI expression in these tumors acts to counteract cell proliferation (Singha et al., 2014; Fournier et al., 2015; Li et al., 2015; Du et al., 2018; Abdelaziz et al., 2019), but this activity is highly dependent on tumor origin (Rae et al., 2001; Giannini et al., 2003; Vo Nguyen et al., 2014; Singha et al., 2019).

TMEPAI exerts its activity, in part, by inhibiting the SMAD2/3 signaling pathway, at the level of the plasma membrane (Watanabe et al., 2010). To date, this action of TMEPAI has only been demonstrated in relation to TGF-β1 signaling (Watanabe et al., 2010; Nakano et al., 2014). In this context, TMEPAI competes with the adaptor protein Smad anchor for receptor activation (SARA) for binding to SMAD2. This competition prevents SARA from recruiting SMAD2 to the TGF-β type I receptor (TβR1/ALK5) and, thereby, reduces the TGF-β1 transcriptional response (Watanabe et al., 2010). TMEPAI exerts this activity via a short SMAD-inhibitory domain (SIM), which lies within its C-terminal cytoplasmic region. Targeted disruption of the SIM domain inhibits the ability of TMEPAI to block TGF-β1-mediated SMAD2/3 signaling. The SIM domain in TMEPAI is flanked by two “PY” motifs (PPXY), which are dispensable for inhibition of TGF-β1 signaling but required for regulation of Akt signaling pathways (Watanabe et al., 2010). Although there is some evidence to support that TMEPAI is capable of also blocking activin A-mediated biological effects (Watanabe et al., 2010), the extent to which TMEPAI can regulate signaling mechanisms in skeletal muscle has not been determined.

In this study we aimed to test the hypothesis that TMEPAI can attenuate myostatin/activin signaling in skeletal muscle fibers to promote hypertrophy and attenuate muscle wasting. We generated an AAV vector expressing TMEPAI and tested its capacity to increase muscle mass following intramuscular injection in healthy mice, and to preserve muscle mass in settings of muscle wasting associated with increased activin A expression. Our studies provide the first evidence that muscle-directed expression of TMEPAI can attenuate the atrophic actions of activin/myostatin activity with beneficial consequences for the maintenance of skeletal muscle mass.

## Materials and Methods

### Generation of TMEPAI Constructs

A pCDNA 3.1 mammalian vector comprising the full length human TMEPAI sequence (reference sequence NM_020182.4) was obtained from GenScript (New Jersey, United States). A C-terminal polyhistidine-tag (8x histidine) was first introduced by PCR, to facilitate downstream purification of TMEPAI. The polyhistidine-tag was incorporated by PCR using an N-terminal TMEPAI primer (sense sequence 5′-CTAGAAGCTT ATGCACCGCTTGATGGGGGTCAACAGCAC-3′) with a C-terminal poly-histidine primer (anti-sense sequence 5′-CTA GGCGGCCGCCTAATGATGGTGGTGATGGTGATGATGGAG AGGGTGTCCTTTCTGTTTATCCTTCTCTTTGCTCCAGATG GC-3′). The primers incorporated restriction sites for *Hind*III and *Not*I, respectively, to enable insertion of the resulting TMEPAI+HIS PCR product into these sites of the pCDNA 3.1 vector. Point mutations in the PY-domains of TMEPAI were generated using the Quik-Change Lightning mutagenesis kit (Agilent Technologies), according to the manufacturer’s guidelines. Primers used for this mutagenesis were as follows: TMEPAI-SIM (sense 5′-GGTGCGCGCACCCGCTGCAAGAAC CATCTTCGAC-3′ and anti-sense 5′-GTCGAAGATGGTTC TTGCAGCGGGTGCGCGCACC-3′), TMEPAI-PY1 (sense 5′-GAGCCCCCACCCGCTCAGGGCCCCTGCAC-3′ and anti- sense 5′-GTGCAGGGGCCCTGAGCGGGTGGGGGCTC-3′), and TMEPAI-PY2 (sense 5′-GCCGCCGCCCACCGCTAGCG AGGTCATCG-3′ and anti-sense 5′-CGATGACCTCGCTAGCG GTGGGCGGCGGC-3′). Introduced mutations were confirmed using DNA sequencing.

### Expression of TMEPAI Variants in HEK293T Cells

Production of TMEPAI protein variants was verified by transient transfection in human embryonic kidney (HEK293T) cells cultured in Dulbecco’s modified Eagle’s medium (DMEM) supplemented with 10% foetal calf serum (FCS), 100 mM sodium pyruvate and 1% penicillin/streptomycin (all reagents from Thermo Fisher Scientific, Massachusetts, United States). For transfection, HEK293T cells were plated at 8 × 10^5^ cells/well in 6-well plates and incubated overnight at 37°C. The following day, 5 μg of TMEPAI construct DNA was combined with Lipofectamine 2000 (Thermo Fisher Scientific) in OPTI-MEM media and added to the cells as outlined by the manufacturer. Following a 24-h incubation, the cells were lysed for western blot analysis (as described below).

### Assessment of TMEPAI Bioactivity *in vitro*

The ability of TMEPAI to block SMAD2/3-mediated ligand signaling was assessed in a HEK293T luciferase bioassay. In brief, HEK293T cells were first seeded into a 48-well plate at a density of 7.5 × 10^4^ cells/well in DMEM containing 10% FCS (without antibiotics). At 24 h post-plating, cells were transfected with the SMAD2/3-responsive A3-Luciferase reporter construct and FAST2 transcription factor (Liu et al., 1999) as well as increasing amounts of either wild-type or mutant TMEPAI constructs (0–9 ng/well), using Lipofectamine 2000 (Thermo Fisher Scientific). At 24 h post-transfection, cells were treated with 200 pM of various SMAD2/3 activating ligands (myostatin, activin A, activin B, and GDF11, all sourced from R&D Systems, Minnesota, United States, or TGF-β1 from PeproTech, United States). The following day, cells were harvested in solubilisation buffer (1% Triton X-100, 25 mM glycylglycine, pH 7.8, 15 mM MgSO4, 4 mM EGTA, and 1 mM dithiothreitol), and the SMAD2/3-induced luciferase activity was determined. Luciferase activity was determined as a % control of the ligand stimulated cells. Ligand treatments typically resulted in a 10- to 30-fold increase in basal luciferase activity. Total Area Under the Curve was compared for mutant TMEPAI forms (SIM and PY1 + PY2) relative to wild type TMEPAI luciferase activities. Values are total area ± standard error. Significance was determined using two-way ANOVAs with Tukey’s *Post Hoc* analysis.

To support this analysis, the inhibition of ligand-mediated phosphorylation of SMAD2 and SMAD3 by the TMEPAI variants was determined by western blot. In brief, HEK293T cells were plated at 4 × 10^5^ cells/well in 12-well plates in DMEM media with 10% FCS (without antibiotics). The following day, the cells were transfected with TMEPAI constructs (0 or 1 μg DNA/well) using Lipofectamine 2000. After a 24-h incubation, cell culture media was replaced with low serum media (DMEM, 0.2% FCS and 50 mM HEPES) and incubated for 4 h at 37°C to suppress basal activation of pSMAD2/3. Cells were then treated with 200 pM activin A or TGF-β1 (diluted in low serum media) and incubated for 45 min. The treated HEK293T cells were subsequently processed for western blotting as described below.

### Western Blotting

HEK293T cells were lysed in ice cold RIPA buffer (10 mM Tris-Cl, 1 mM EDTA, 0.5 mM EGTA, 1% Triton X-100, 0.1% sodium deoxycholate, 0.1% SDS and 140 mM NaCl, pH 8.0) containing Complete protease inhibitors and PhosStop phosphatase inhibitors (both Roche Applied Sciences). Skeletal muscles were homogenized in ice cold lysis buffer containing 50 mM Tris, pH 7.5, 150 mM NaCl, 5 mM MgCl_2_, 10% glycerol, 1% SDS, 1% Triton X-100, and phosphatase (P5726, Sigma Aldrich, Missouri, United States) and protease (P8340, Sigma) inhibitors. Following lysis, samples were rotated for 20 min at 4°C, incubated at 70°C for 10 min with frequent mixing and centrifuged at 18,000 *g* for 20 min at 4°C. The protein concentration of resulting supernatants was determined using a BCA protein assay (Thermo Fisher Scientific), samples were reduced with a final concentration of 2.5% β-mercaptoethanol and then denatured for 5 min at 95°C. Protein fractions were resolved by SDS-PAGE using pre-cast 10% Tris-Glycine (Bio-Rad, California, United States) or 4–12% Bis-Tris gels (Thermo Fisher Scientific), blotted onto nitrocellulose or PVDF membranes (Bio-Rad), blocked for a minimum of 1 h in either 5% skim milk powder or 1% BSA in TBS-T (50 mM Tris, 150 mM NaCl, 0.05% Tween-20, pH 8.0) and incubated overnight at 4°C in primary antibody solutions. All primary antibodies were used at a dilution of 1:1000 unless otherwise stated. Antibodies against pSmad2^*Ser465/467*^ (#3101), total SMAD2/3 (#8685), and GAPDH-HRP (#3683) were purchased from Cell Signaling Technologies (Massachusetts, United States). Antibodies targeting pSmad3^*Ser423*/425^ (#ab52903) were purchased from Abcam (Cambridge, United Kingdom). Antibodies targeting Filamin C (1:2,000) were purchased from Sigma Aldrich. TMEPAI was detected using an anti-PMEPA1/TMEPAI antibody (#85829 Santa Cruz Biotechnology, United States) at 1:1000. Bound primary antibodies were detected by incubation with HRP-conjugated secondary rabbit (#7074, Cell Signaling Technologies), mouse (#NXA931V, Amersham, United Kingdom), or goat antibodies (#P0448, Agilent/Dako, United States) in 5% skim milk powder or 1% TBS-T for 1 h. Chemiluminescence was detected using ECL western blotting detection reagents (GE Healthcare, Buckinghamshire, United Kingdom) and Immobilon Forte western HRP substrate (Merck, New Jersey, United States).

### Production of AAV Vectors

cDNA constructs encoding TMEPAI or activin A (ActA) were cloned into AAV expression plasmids consisting of a cytomegalovirus (CMV) or the tetracycline response element (TRE) promoter and SV40 poly-A region flanked by AAV2 terminal repeats. Viral vector production was performed as described previously (Blankinship et al., 2004). Briefly, HEK-293 cells were plated at a density of 7.2–8.5 × 10^6^ cells on a 15 cm culture dish and incubated for 8–16 h. The cells were then transfected with 22.5 μg of a vector genome-containing plasmid and 45 μg of the packaging/helper plasmid pDGM6 using calcium phosphate precipitation. At 72 h post transfection, the media and cells were collected and lysed via three freeze-thaw cycles before 0.22 μm clarification (Merck). Purification of viral vector particles from crude lysates was performed using affinity chromatography via heparin affinity column (HiTrap, Amersham) and ultracentrifugation overnight prior to re-suspension in sterile physiological Ringer’s solution. The purified vector preparations were titered with a customized sequence-specific quantitative PCR-based reaction (Thermo Fisher Scientific).

### Animal Experiments

All experiments were conducted in accordance with the relevant code of practice for the care and use of animals for scientific purposes (National Health and Medical Council of Australia). All experimental protocols were approved by the Alfred Medical Research and Education Precinct Animal Ethics Committee (AMREP AEC) and University of Melbourne Animal Ethics committee. Cohorts of 8-week-old C57Bl/6J mice were obtained from the breeding colony maintained by the AMREP Animal Services facility (Melbourne, Australia), the founders of which were sourced from the Jackson Laboratory. Cohorts of 7-week-old BALB/c mice were sourced from the Walter and Eliza Hall Institute Bioservices Kew Division. Mice were fed standard chow diets unless otherwise stated, with access to drinking water *ad libitum* while housed under a 12:12-h light dark cycle. All surgical procedures were performed on mice placed under general anesthesia via inhalation of isoflurane in medical oxygen supported by post-operative analgesia. AAV doses administered to mice were determined from dose optimization experiments. AAV:TMEPAI was administered to mice at a dose of 1 × 10^9^ vg. For studies involving the over-expression of activin A, a tetracycline-inducible gene expression construct (AAV:TetOn) and a TetOn-responsive ActA (AAV:TRE-ActA) construct were administered at doses of 1 × 10^10^ vg and 1 × 10^9^ vg, respectively. Control treatments consisted of delivery of a viral vector lacking a functional gene (Con) into the contralateral limb at equivalent vector genome doses. Vectors were diluted in 30 μl of Hank’s buffered saline solution and injected into the anterior compartments of the lower hind limb of anesthetized mice. Following AAV:TetOn and AAV:TRE-ActA administration, mice were provided standard chow containing 600 mg/kg doxycycline (Specialty Feeds), to facilitate sustained gene expression. C57Bl/6J mice administered AAV:TRE-ActA, AAV:TetOn and AAV:TMEPAI were analyzed 4 weeks after treatment. Implantation of C26 solid tumor tissue into the flank of BALB/c mice was performed, as previously described (Aulino et al., 2010; Winbanks et al., 2016). Tumor-bearing mice were treated with AAV:TMEPAI and AAV:Control at the time of tumor implantation. The experimental endpoint was determined by an ethical criterion of ∼25% loss of initial body mass. At the experimental endpoint, mice were humanely euthanized via overdose of sodium pentobarbitone (100 mg/kg) or cervical dislocation. Skeletal muscles, tissues and organs were excised rapidly and weighed before subsequent processing.

### Skeletal Muscle Histology

Portions of harvested muscles were placed in OCT cryoprotectant (ProSciTech, Queensland, Australia) and frozen in liquid nitrogen-cooled isopentane. Frozen samples were cryosectioned at 10 μm thickness for hematoxylin and eosin staining as previously described (Hagg et al., 2016). Sections were mounted using DePeX mounting medium (VWR, Leicestershire, England) and imaged at room temperature using a U-TV1X-2 camera mounted to an IX71 microscope and a PlanC 10X/0.25 objective lens (Olympus). Images of sections were obtained using acquisition software (DP2-BSW, Olympus). Analysis of myofibre diameter was performed on muscle samples cryosectioned at 8 μm thickness. Sections were incubated in FITC blocking buffer (Thermo) for 30 min and then in Alexa Fluor 555-conjugated Wheat Germ Agglutinin (WGA; diluted in PBS) for 2 h. Sections were stained with DAPI, mounted in Mowiol 4-88 and imaged using a fluorescence microscope (Axio Imager M2, Zeiss). The minimum Feret’s diameter of muscle fibers was determined by measuring at least 400 fibers per mouse muscle, using ImageJ software (United States National Institutes of Health, Bethesda, MD, United States).

### Quantitative PCR Analysis of Target Genes

Total RNA was isolated from skeletal muscles using TRIzol (Thermo Fisher Scientific) according to manufacturer’s instructions. First, 1 μg of RNA was reverse transcribed using the High Capacity RNA-to-cDNA kit (Thermo Fisher Scientific). Gene expression was analyzed by qRT-PCR using SYBR primers (Sigma and Thermo Fisher Scientific) and Real-Time PCR detection (CFX384, Bio-Rad). Target gene expression was normalized to *Hprt*. Data were analyzed using the ΔΔCT method of analysis and are presented as fold change of the control sample (expressed as 1). Oligonucleotide primer sequences used are listed in Table 1.

**TABLE 1:** Oligonucleotide sequences used to perform qRT-PCR.

**Gene**	**Fwd sequence**	**Rev sequence**
*mTmepai*	GAAGGCAAAAGAGAAAATGC	GAAATTAAGCATTCACGCAC
*mIgfn1*	CCTCATTGTCACAGAATACG	ATTTGCCATCCATCTCATAG
*mCyr61*	AGAGGCTTCCTGTCTTTG	GTTGTCATTGGTAACTCGTG
*mMurf1*	ACCTGCTGGTGGAAAACATC	CTTCGTGTTCCTTGCACATC
*mAtrogin1*	GCAAACACTGCCACATTCTCTC	CTTGAGGGGAAAGTGAGACG
*mHprt*	GTTTGTTGTTGGATATGCCCTTG	GGCAACATCAACAGGACTCC

### Experimental Design and Statistical Analysis

*In vitro* studies were replicated three times. *In vivo* studies were performed once. Exclusion criteria for animals were applied in the case of death, cannibalism or tumor ulceration. Exclusion criteria for samples were applied in the case of histological artifacts (freeze- and sectioning-damage) and RNA or protein degradation. One- and two-way ANOVAs were used to assess statistical differences between more than two conditions, with the Tukey’s *post-hoc* test used for comparisons between the specific group means (GraphPad Prism v.6, La Jolla, CA, United States). Data are presented as the means ± SEM. Comparisons between two conditions utilized the Student’s *t*-test. Statistically significant changes are denoted by asterisks, level of significance is outlined in individual figure legends. Significance denoted by ^****^p < 0.0001, ^∗∗∗^*p* < 0.001, ^∗∗^*p* < 0.01, and ^∗^*p* < 0.05.

## Results

### TMEPAI Inhibits the *in vitro* Activity of Multiple SMAD2/3-Activating Ligands

In previous studies conducted by our laboratories, transcriptional profiling indicated that skeletal muscle expression of TMEPAI mRNA (*Pmepa1*) increased following exposure to activin A (Chen et al., 2014) and decreased when activin and/or myostatin signaling pathways were inhibited (Chen et al., 2017). In this study, qPCR analysis of mouse tibialis anterior (TA) muscles injected with AAV:ActA confirmed that TMEPAI transcription increased markedly (6-fold) in response to activin A expression (Supplementary Figure 1A). This evidence supports the hypothesis that activin A, like TGF-β1, can transcriptionally activate TMEPAI, likely via their shared SMAD2/3 intracellular pathway.

**FIGURE 1:**
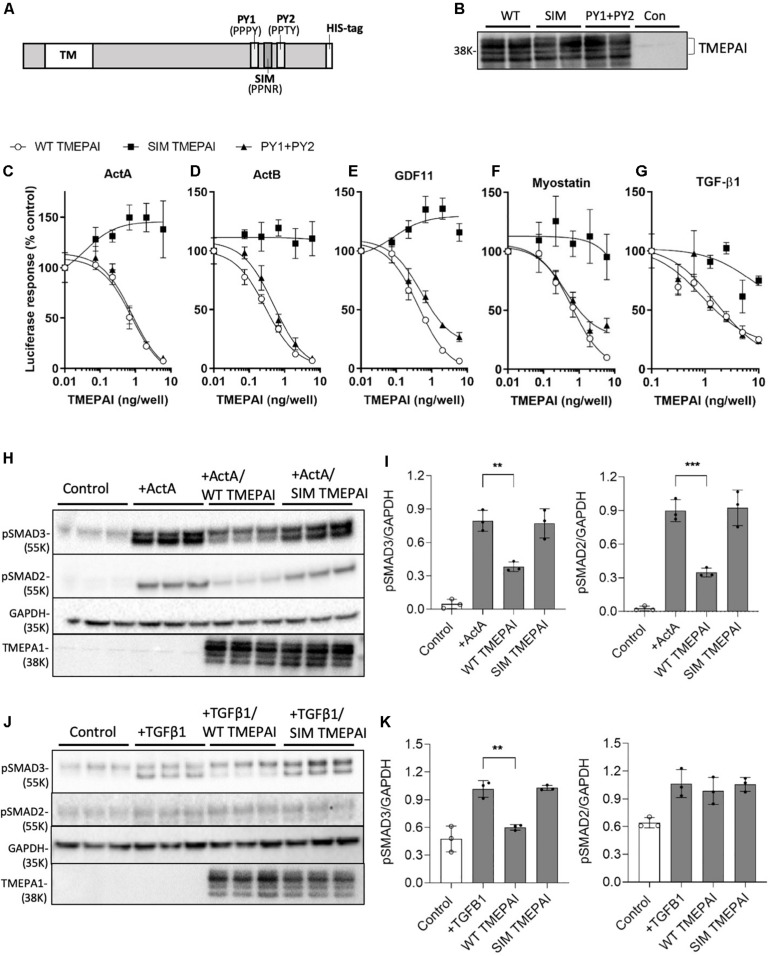
TMEPAI inhibits the *in vitro* activity of multiple SMAD2/3 ligands via its SIM domain. **(A)** Schematic model of TMEPAI noting transmembrane (TM), SMAD2/3 inhibitory domain, pAkt PY motifs (PY) and placement of an inserted polyhistidine (HIS) tag. **(B)** Expression of TMEPAI variants in transfected HEK293T mammalian cells. Reduced cell lysates were probed with an anti-TMEPAI antibody. Multiple TMEPAI protein species are likely owing to differential posttranslational modifications. **(C–G)** The ability of TMEPAI to block ligands that signal through SMAD2/3 was determined using a SMAD2/3 responsive luciferase reporter assay in HEK293T cells. Cells were transfected with plasmids encoding for the luciferase reporter and increasing doses of TMEPAI, then stimulated with the ligands **(C)** activin A, **(D)** activin B, **(E)** GDF-11, **(F)** Myostatin, and **(G)** TGF-β1. Luciferase activity was determined as a measure of SMAD2/3 induction (*n* = 3 samples/treatment, representative result of *n* = 3 replicates). **(H, I)** Western blot analysis of HEK293T lysates revealing that inactivation of the SIM domain in TMEPAI inhibits the ability of TMEPAI to block activin A-induced endogenous SMAD2 and SMAD3 phosphorylation. **(J, K)** TMEPAI also blocks TGFβ1-induced endogenous SMAD3 phosphorylation, but not SMAD2 in HEK293T cells. For western blots, *n* = 3 samples/treatment condition, ***p < 0.001 and **p < 0.01.

TMEPAI provides negative feedback for TGF-β1-mediated SMAD2/3 signaling (Watanabe et al., 2010; Nakano et al., 2014), however, its ability to restrict the activity of related SMAD2/3 activators has not been explored. To test this hypothesis, we transfected cells with constructs expressing wild-type TMEPAI and versions containing mutations in the SIM and PY1/PY2 domains implicated in protein-protein interactions, (Figures 1A,B) and examined SMAD2/3 signaling activity in response to ligand exposure (Figures 1C–G). We found that TMEPAI also suppresses SMAD2/3-induced luciferase expression triggered by myostatin, activin A, activin B, and GDF-11, in addition to TGF-β1 (Figures 1C–G). Transient transfection of cells with constructs expressing TMEPAI (Figure 1B) exerted inhibitory activity via the tryptophan-rich SIM domain (Figure 1A), as mutation of this region (PPNR to PPAA) abolished the ability of TMEPAI to block luciferase activity induced by all ligands tested (Figures 1C–G and Table 2). In contrast, flanking PY1/PY2 motifs within TMEPAI, thought to mediate interactions with the ubiquitin ligase NEDD4 (Watanabe et al., 2010), were not required for TMEPAI inhibitory activity against these ligands (Figures 1C–G). Mechanistically, TMEPAI blocks both endogenous SMAD2 and SMAD3 pathways, as shown by suppression of activin A-induced SMAD2/3 phosphorylation in HEK293T cells (Figures 1H,I). TMEPAI also blocked TGFβ1-induced SMAD3 phosphorylation in HEK293T cells, however, the cells were unresponsive to TGFβ1-induced SMAD2 activation (Figures 1H–K). These findings demonstrate that TMEPAI can inhibit SMAD2/3 activation by myostatin, activin A, activin B and GDF-11 via protein interactions dependent on the TMEPAI SIM domain.

**TABLE 2:** Luciferase assay data – Area Under the Curve analyses.

	**WT TMEPAI**	**SIM TMEPAI**	**PY1 + PY2**
Activin A	192.1 ± 7.9	368 ± 12.8****	202 ± 9*^*ns*^*
Activin B	147.2 ± 6.1	307.8 ± 8.9****	177 ± 6*^*ns*^*
GDF11	169.1 ± 4.8	328.9 ± 6.1****	205 ± 4*^*ns*^*
Myostatin	184.4 ± 5.1	304.3 ± 14.3***	199 ± 9*^*ns*^*
TGF-β1	117.7 ± 18.2	178.9 ± 9.1***	126 ± 6*^*ns*^*

Total Area Under the Curve (arbitrary values) was compared for mutant TMEPAI forms (SIM and PY1 + PY2) relative to wild type TMEPAI. Values are total area ± standard error. Significance was determined using two-way ANOVAs with Tukey’s *Post Hoc* analysis (*n* = 3 samples). ****p < 0.0001, ***p < 0.001, and *ns* = not significant.

### TMEPAI Promotes Skeletal Muscle Hypertrophy and Blocks Activin A-Mediated Muscle Wasting *in vivo*

**FIGURE 2:**
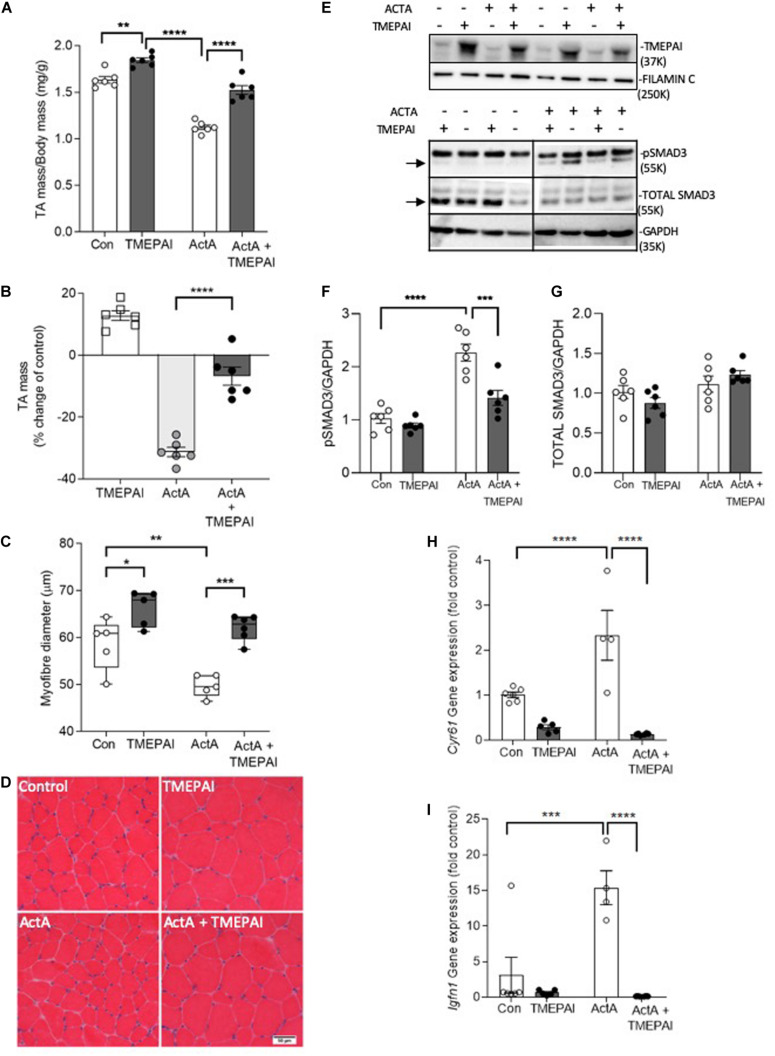
TMEPAI promotes skeletal muscle hypertrophy and blocks activin A-mediated muscle wasting *in vivo*. **(A, B)** 4 weeks after the TA muscles of 6–8-week-old C57Bl/6J were injected with AAV vectors encoding TMEPAI and/or activin A (or equivalent doses of AAV lacking the transgene), muscles were excised to measure mass (*n* = 6 samples per treatment condition). **(C, D)** Mean muscle fiber diameter and representative morphology according to haematoxylin and eosin staining (*n* = 5 samples per treatment condition). **(E)** Protein lysates of muscles were subjected to western blot analysis of intracellular target pSMAD3 and **(F, G)** protein abundance was quantified using densitometric methods (*n* = 6 samples per treatment condition). **(H, I)** Expression of SMAD2/3-target genes *Cyr61* and *Igfn1* in muscles (*n* = 4–6 per treatment group). ****p < 0.0001, ***p < 0.001, **p < 0.01, and *p < 0.05.

Basal SMAD2/3 activation by endogenous myostatin and activins restricts muscle growth (Chen et al., 2017). Additionally, overexpression of myostatin, activin A, and activin B, but not TGF-β1, induces muscle atrophy (Chen et al., 2014). Therefore, we sought to test the hypothesis that increased expression of TMEPAI in muscle would counteract the ligands that negatively regulate muscle mass via SMAD2/3 activation. Here, we found that delivery of AAV:TMEPAI to the TA hindlimb muscles of mice promoted muscle hypertrophy, as demonstrated by a 12% increase in TA mass (Figures 2A,B) and 16% increase in muscle fiber size (Figures 2C,D). We have shown that elevated circulating levels of activin A, similar to those observed in patients with cancer cachexia (Loumaye et al., 2015) and in mouse models of cancer cachexia, can induce profound muscle wasting (Chen et al., 2014). Therefore, we examined whether TMEPAI could protect skeletal muscle against activin A-induced atrophy. Examination of C57Bl/6J male mice 4 weeks after intramuscular injection of an AAV vector expressing activin A (AAV:ActA) identified a 30% reduction in TA muscle mass compared with contralateral muscles (Figures 2A,B). In contrast, co-delivery of AAV:TMEPAI with AAV:ActA attenuated activin A-induced loss of muscle mass (Figures 2A,B). Histological analysis indicated that TMEPAI protected muscle fibers from activin A-mediated atrophy (Figures 2C,D). In terms of mechanism, we analyzed SMAD3 phosphorylation in the TA muscles of mice expressing activin A in the presence, or absence, of TMEPAI. Western blot analysis of muscle lysates indicated that TMEPAI suppressed activin A-mediated phosphorylation of pSMAD3 (Figures 2E–G). Additionally, TMEPAI potently inhibited activin-induced upregulation of SMAD2/3-target genes *Cyr61* and *Igfn1* (Figures 2H,I). Together, these *in vivo* studies demonstrate that TMEPAI inhibits the SMAD3-mediated transcriptional response initiated by exogenous, activin A, thereby, protecting muscle mass.

### TMEPAI Preserves Muscle Mass in a Mouse Model of Cancer Cachexia

**FIGURE 3:**
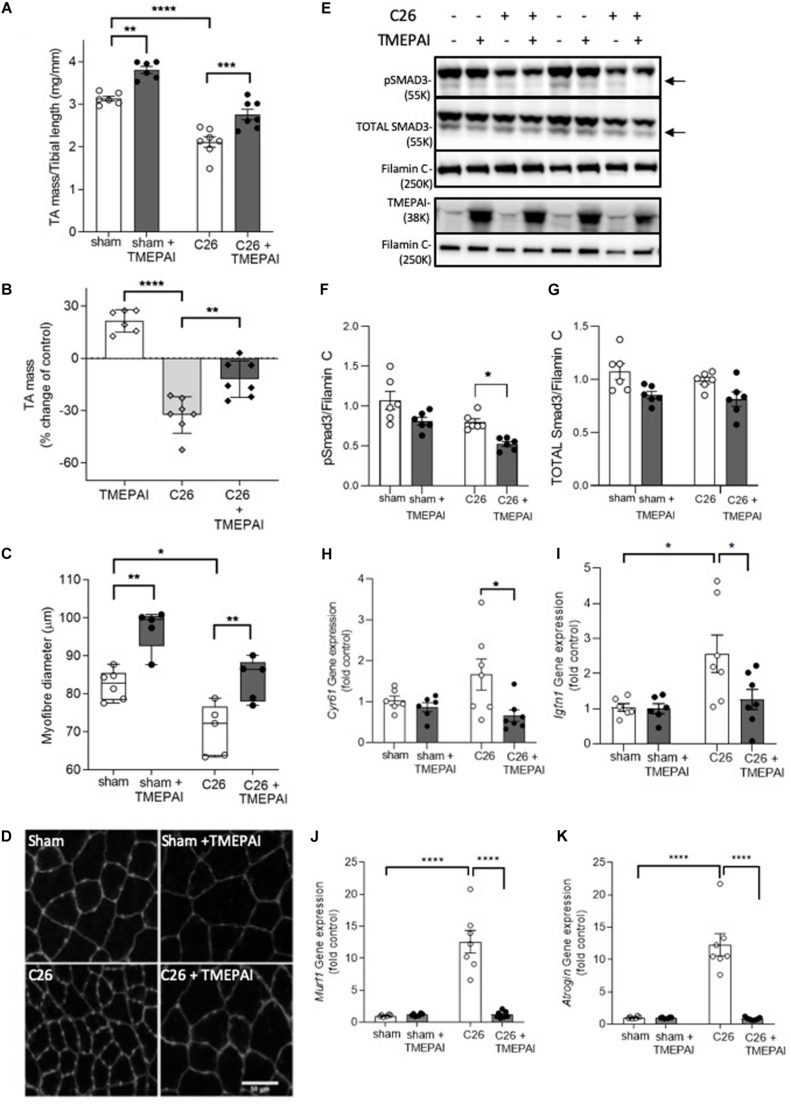
TMEPAI prevents localized muscle wasting in a mouse model of cancer cachexia. **(A, B)** 7-week-old BALB/c mice implanted with subcutaneous Colon-26 tumors (or subjected to sham surgery) were administered TA muscle injections of AAV vector expressing TMEPAI (or control vector). At experimental endpoint, TA muscles were harvested and weighed (*n* = 6–7 samples per treatment group). **(C, D)** Muscle fiber size and representative morphology indicated by wheat germ agglutinin labeling of TA muscle cryosections (*n* = 5 samples per treatment group). **(E)** Western blot analysis of muscle protein lysates for assessment of SMAD3 phosphorylation, and **(F, G)** protein abundance quantified by densitometry (*n* = 6–7 samples per treatment group). **(H, I)** The transcription of SMAD2/3-target genes and **(J,K)** key muscle atrophy-related genes in the muscles from the aforementioned mice (*n* = 6–7 samples per treatment group). ****p < 0.0001, ***p < 0.001, **p < 0.01, and *p < 0.05.

As myostatin and activins have been implicated in the pathogenesis of cancer cachexia, we next sought to determine if TMEPAI could prevent loss of muscle mass in a mouse model of cancer cachexia, induced by the subcutaneous growth of an implanted colon-26 (C26) carcinoma in male BALB/c mice (Aulino et al., 2010; Zhou et al., 2010; Winbanks et al., 2016). In the absence of tumor growth, intramuscular injection of AAV:TMEPAI promoted a significant increase in TA mass (21%) and fiber size (20%) in BALB/c mice (Figures 3A,B), which was similar to the changes observed in C57Bl/6J mice (Figures 2A,B). BALB/c mice bearing C26 tumors displayed a 32% reduction in TA mass, however, intramuscular administration of TMEPAI significantly attenuated muscle loss (Figures 3A,B). TMEPAI-expressing TA muscles in C26 tumor-bearing mice were on average only 12% reduced in mass when compared with the muscles of tumor-free mice (Figures 3A,B). Histological analysis supported these findings, demonstrating a greater average fiber diameter in muscles from C26 tumor-bearing mice administered AAV:TMEPAI, compared to contralateral limb muscles administered AAV:Control (Figures 3C,D). This average myofibre size was larger than that observed in the TA muscles of tumor-free control mice (Figures 3C,D). Phosphorylation of SMAD3 was significantly reduced in the presence of TMEPAI in the muscles of C26 tumor-bearing mice (Figures 3E–G), and TMEPAI expression also attenuated transcriptional activation of SMAD2/3-target genes in C26 tumor-bearing mice, including *Cyr61*, and *Igfn1* (Figures 3H,I). Additionally, TMEPAI expression suppressed transcription of the muscle atrophy genes *MuRF-1* and *Atrogin-1* (Figures 3J,K).

## Discussion

Due to their potent actions on muscle and other tissues, ligands that signal via activin type II receptors are amongst the most highly regulated members of the TGF-β superfamily. Myostatin is secreted in a latent complex with its prodomain and requires activation by tolloid metalloproteases (Wolfman et al., 2003). However, activation alone does not guarantee receptor binding, as numerous extracellular binding proteins, including follistatin, follistatin-like 3, GASP-1 and GASP-2, inhibit myostatin activity (Chen et al., 2016a). While activin A and activin B are secreted in active forms, they too are neutralized by follistatin and follistatin-related proteins (Nakamura et al., 1990; Tsuchida et al., 2000) and receptor access can be blocked by inhibin A and inhibin B (Lewis et al., 2000). Many of these antagonists and soluble forms of the activin type II receptor have been shown to increase muscle mass in healthy mice and mice modeling muscle wasting conditions (Li et al., 2007; Zhou et al., 2010; Winbanks et al., 2012; Parente et al., 2020). However, the potential side effects of molecules that target ligands with broad expression and cell tropism such as activin A/B (Walton et al., 2012; Campbell et al., 2017) may limit their therapeutic utility.

An alternative approach to inhibit myostatin/activin activity is to limit SMAD2/3 phosphorylation downstream of activin type I receptors in cells of interest. We previously demonstrated the potential of this approach by using AAV vectors to increase expression of SMAD7 in skeletal muscle fibers (Winbanks et al., 2016). SMAD7 prevents SMAD2/3 phosphorylation, promotes ActRIIB degradation (Hayashi et al., 1997), and was observed to increase muscle mass in healthy mice and attenuate wasting in models of cachexia (Winbanks et al., 2016). Other SMAD2/3 inhibitory proteins, including Smurf, c-Ski, SnoN, TGIF, and TMEPAI, have primarily been studied in the context of TGF-β1 signaling (Akiyoshi et al., 1999; Stroschein et al., 1999; Wotton et al., 1999; Zhu et al., 1999). However, the recent observation that activin induces TMEPAI expression in skeletal muscle (Chen et al., 2014) suggested that this membrane-associated protein may be involved in an intracellular negative feedback system that limits the activation of SMAD2/3 and, thereby, helps maintain muscle homeostasis.

TMEPAI is a membrane- or lysosome-associated protein that regulates not only TGF-β1 signaling but also androgen receptor (AR) and phosphatase and tensin homologue deleted on chromosome 10 (PTEN) activity (Watanabe et al., 2010; Singha et al., 2014). Regulation of these pathways help to explain a link between TMEPAI and tumourigenicity, where it has been shown TMEPAI can either limit cancer progression and metastases (Singha et al., 2014; Fournier et al., 2015; Li et al., 2015; Du et al., 2018; Abdelaziz et al., 2019) or promote cancer development (Rae et al., 2001; Giannini et al., 2003; Vo Nguyen et al., 2014; Singha et al., 2019). In this study, we demonstrated that TMEPAI also inhibits myostatin, activin A, activin B and GDF11 signaling, revealing that TMEPAI operates as a general inhibitor of SMAD2/3-activating ligands in muscle. Overexpression of TMEPAI specifically in skeletal muscle enhanced muscle mass by increasing fiber size and abrogated muscle atrophy induced by overexpression of activin A. Moreover, TMEPAI expression attenuated muscle wasting in an established mouse model of cancer cachexia.

In muscles overexpressing activin A, TMEPAI inhibits SMAD3 (and potentially SMAD2) phosphorylation and reduces the activin transcriptional response, as evident by a significant decrease in the expression of activin-responsive genes (e.g., *Cyr61* and *Igfn1*). Similarly, in the muscles of C26 tumor-bearing mice, where activin A serum concentrations are increased (Chen et al., 2016b), SMAD3 phosphorylation is significantly reduced by TMEPAI. In support, expression of SMAD2/3 target genes *Cyr61* and *Igfn1* is also reduced in the muscles of C26 tumor-bearing mice in response to TMEPAI expression. Further, in tumor-bearing mice, TMEPAI expression suppressed transcriptional activation of muscle atrophy genes, *Murf-1* and *Atrogin-1*, likely contributing to its protective effects against muscle wasting. However, as TMEPAI can also activate SMAD-independent AR and PTEN/Akt signaling pathways (Itoh and Itoh, 2018), it is possible that additional mechanisms may contribute to TMEPAI-mediated muscle hypertrophy in these mice. Mechanistically, we showed that TMEPAI exerted its inhibitory activity via a short SIM domain, which lies within its C-terminal cytoplasmic region. Targeted disruption of the SIM domain inhibited the ability of TMEPAI to block activin A, activin B, myostatin and GDF11 activity. Conversely, the flanking PY motifs were dispensable for inhibition of SMAD2/3 signaling. This is important because the PY motifs mediate the interaction of TMEPAI with other signaling pathways (Watanabe et al., 2010) and suggest that the TMEPAI (PY) variant could be utilized to specifically inhibit SMAD2/3 signaling in skeletal muscle.

The data presented here demonstrate that TMEPAI gene delivery to skeletal muscle can attenuate activin signaling and preserve or increase muscle mass. As inhibition of activin signaling has also been shown to positively regulate metabolic processes (Han et al., 2019; Davey et al., 2020), expression of TMEPAI may also confer benefits in settings of metabolic disease. The signaling actions of TMEPAI within muscle fibers suggest that a muscle-restricted intervention could avoid the off-target effects of extracellular ligand traps, such as soluble ActRIIB. The findings support future studies to explore the extent to which systemically administered interventions that increase TMEPAI expression in skeletal muscles can protect against body-wide muscle wasting in different disease settings.

## Data Availability Statement

The raw data supporting the conclusions of this article will be made available by the authors, without undue reservation.

## Ethics Statement

The animal studies were reviewed and approved by the Alfred Medical and Education Precinct Animals Ethics Committee (AMREP AEC), and the University of Melbourne Animal Ethics Committee.

## Author Contributions

AH, PG, CH, and KW designed the experiments. AH, SK, GG, CG, JC, RT, and HQ undertook the experiments. AH, PG, CH, and KW analyzed the data. AH, PG, CH, and KW prepared the manuscript. All authors had the opportunity to review the final version of the manuscript.

## Conflict of Interest

The authors declare that the research was conducted in the absence of any commercial or financial relationships that could be construed as a potential conflict of interest.

## Supplementary Material

Supplementary Figure 1 TMEPAI expression in activin A expressing muscles. **(A)** TMEPAI (*Pmepa1*) gene expression was measured in muscles previously injected with AAV:ActivinA (*n* = 5–6), ^∗∗^*p* < 0.01.
