# Supplementary figures and images for: TMEPAI/PMEPA1 Is a Positive Regulator of Skeletal Muscle Mass
Source: Front Physiol. 2020 Nov 4;11:560225. doi: 10.3389/fphys.2020.560225 (PMC7672205; doi:10.3389/fphys.2020.560225)

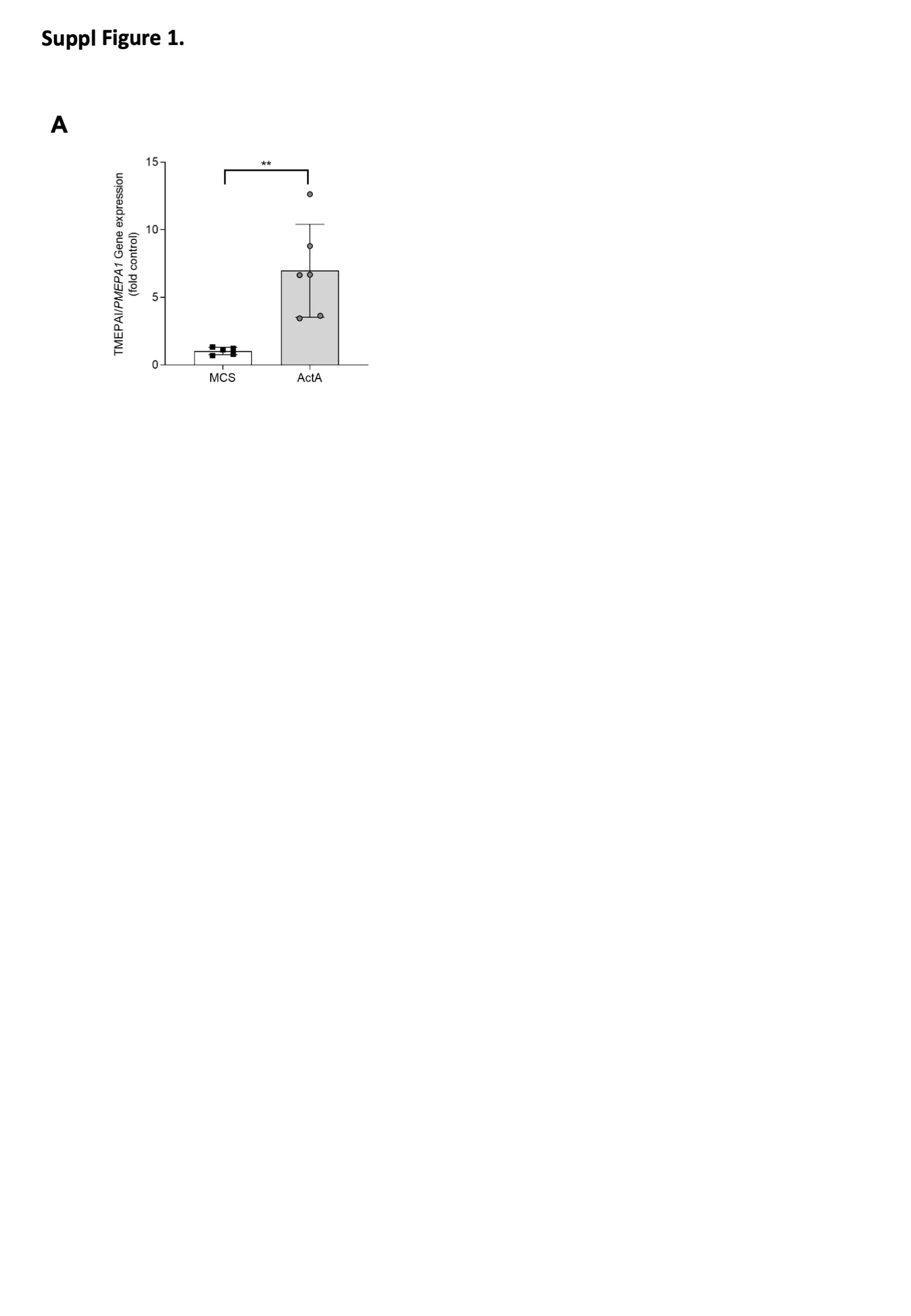

Supplement: Supplementary Figure 1 — TMEPAI expression in activin A expressing muscles. (A) TMEPAI (Pmepa1) gene expression was measured in muscles previously injected with AAV:ActivinA (n = 5–6), ∗∗p < 0.01. [file Figure_1.JPEG]
